# Cohort trends in intrinsic capacity in England and China

**DOI:** 10.1038/s43587-024-00741-w

**Published:** 2024-12-19

**Authors:** John R. Beard, Katja Hanewald, Yafei Si, Jotheeswaran Amuthavalli Thiyagarajan, Dario Moreno-Agostino

**Affiliations:** 1 Robert N. Butler Columbia Aging Center, Columbia University, New York, NY USA; 2 School of Risk & Actuarial Studies, UNSW Sydney, Sydney, New South Wales Australia; 3 ARC Centre of Excellence in Population Ageing Research (CEPAR), Sydney, New South Wales Australia; 4 Ageing and Health Unit, Department of Maternal, Child, Adolescent Health and Ageing, World Health Organization, Geneva, Switzerland; 5 Centre for Longitudinal Studies, UCL Social Research Institute, University College London, London, UK; 6 ESRC Centre for Society and Mental Health, King’s College London, London, UK

**Keywords:** Psychology, Policy, Ageing

## Abstract

To understand how the health of older adults today compares to that of previous generations, we estimated intrinsic capacity and subdomains of cognitive, locomotor, sensory, psychological and vitality capacities in participants of the English Longitudinal Study of Ageing and the China Health and Retirement Longitudinal Study. Applying multilevel growth curve models, we found that more recent cohorts entered older ages with higher levels of capacity, while subsequent age-related declines were somewhat compressed compared to earlier cohorts. Trends were most evident for the cognitive, locomotor and vitality capacities. Improvements were large, with the greatest gains being in the most recent cohorts. For example, a 68-year-old participant of the English Longitudinal Study of Ageing born in 1950 had higher capacity than a 62-year-old born 10 years earlier. Trends were similar for men and women and were generally consistent across English and Chinese cohorts. Possible causes include broad societal influences and improvements in medical care.

## Abstract (web summary)

Intrinsic capacity includes a person’s mental and physical capacities. The authors examined cohort trends in intrinsic capacity in two large English and Chinese studies. They report that more recently born participants entered older ages with markedly higher levels of functioning, while subsequent age-related declines were somewhat delayed.

## Main

Over the past century, life expectancy has risen in almost every country, and longer lives are increasingly becoming the norm^1^. Initially, this trend was driven by increased survival through childhood and childbirth, but in more developed countries, it is now mainly a consequence of longer survival at older ages^2^. Yet, while these improvements in life expectancy are well documented, it is still uncertain how the health of older adults today compares to that experienced by previous generations^3–6^.

One reason for this uncertainty is that health is a multifaceted concept and no consensus has been reached on how to frame or measure it^7^. Temporal trends are complex and multidirectional; they are particularly difficult to understand when considered from the perspective of disease^6^. Broader access to effective healthcare means that some people who would have previously died from a condition now survive into older ages. While this is a positive trend, it results in increased disease prevalence in older age groups^6^. On the other hand, advances in medical care that increase survival may have reduced the impact that these conditions have on people’s lives. New treatments may have also lessened the influence of other conditions. For example, a person with osteoarthritis of the hip, who might have previously experienced severe disability, may now regain high levels of locomotor capacity after joint replacement^8^. Moreover, aging is associated with complex and dynamic biological changes that are often expressed as declines in physical or mental capacity even in the absence of disease^9^. Changes in disease prevalence can tell us little about trends in these underlying dynamics or their broader functional consequences.

Critically, older people often report that the health outcome they value most is not the presence or absence of disease, or even life extension, but their level of functioning and independence^10^. This is also a powerful determinant of their social engagement and economic performance. Therefore, a more relevant way to consider changing patterns of health status might be to examine trends in functioning. However, research into functional trends has been challenging. For example, the Global Burden of Disease group has estimated trends in functioning by applying disability weightings to trends in disease prevalence. However, these disability weights are only indirect, generic estimates of a disease’s impact and cannot account for geographical or temporal variations in healthcare or the many other factors that may mitigate the functional consequences of these conditions^11^.

Most research that has directly estimated functional trends has been limited to measures of severe disability, such as activities of daily living (ADLs) or instrumental activities of daily living (IADLs). The findings of these studies have been varied and inconclusive^12–16^. Furthermore, as loss of ADLs or IADLs is only apparent after very large declines in functioning, these categorical outcomes tell us little about changes in the functional status of the far broader population of older adults who have not experienced these major losses.

Research examining functional trends in these more robust individuals has generally been limited to single aspects of functioning. For example, there is a growing body of work examining trends in cognitive capacity^17,18^. Other analyses have used self-reported measures or components of these measures. However, these analyses cannot provide a comprehensive and objective portrayal of how the overall health of older adults may have changed over time. For example, changes in mental functioning may, or may not, have been accompanied by changes in physical functioning, and self-rated health may be influenced by changing expectations.

A final body of research has examined trends in health status by aggregating a wide range of health deficits operating at different levels, for example, biological measures, risk factors, specific conditions and measures of severe disability. Again the results are somewhat inconsistent^19,20^. These assessments, often called frailty indices, have been used clinically to identify individuals at risk or as surrogate markers of health in some economic analyses. However, they lack a conceptual frame that might account for the hierarchy of, and relationships between, deficits. Most take a summative approach that does not account for the correlations between these characteristics; many of the limitations described in the previous paragraphs also apply to frailty indices.

This article describes an alternative approach to determining health trends based on a recent model of healthy aging proposed by the World Health Organization (WHO). In this strengths-based model, healthy aging is considered not from the perspective of the presence or absence of disease but based on an individual’s ability to be and do the things they value^1^. This ability is understood to be determined by individual-level attributes—a person’s ‘intrinsic capacity’, the environments they inhabit and the interaction between the individual and these environments^21^. Thus, intrinsic capacity is an individual-level construct including all the physical and mental capacities of an individual framed as a continuum that can be considered across the whole of the second half of life^1^. Approaching health from this functional perspective allows an estimation of the health state of the individual that is independent of the presence or absence of disease. This state will reflect both underlying changes in age-related biology and interventions (for example, hip replacement) that mitigate the impact of disease (for example, osteoarthritis).

We have previously determined and validated a measurable structure for intrinsic capacity in two large longitudinal studies of the English and Chinese populations: the English Longitudinal Study of Ageing (ELSA)^9^ and the China Health and Retirement Longitudinal Study (CHARLS)^22^. We considered all variables within these datasets that might contribute to a person’s functioning, limiting our analysis where possible to objective measures, although we also included self-reported assessments of hearing and vision, and the Center for Epidemiological Studies-Depression Scale. We used exploratory factor analysis, bifactor analysis and structural equation modeling to take account of the complex correlations between related variables (for example, chair stand and gait speed). Both analyses identified an intrinsic capacity construct including subdomains of cognitive, locomotor, sensory and psychological capacity and a further capacity subdomain labeled vitality, which may represent underlying age-related biological changes and energy balance. The structure of this construct is consistent with what had been previously suggested by gerontological theory^23^. Our analyses showed intrinsic capacity to have strong construct validity and to be a powerful predictor of subsequent care dependence, even after adjustment for multimorbidity, age, sex and socioeconomic status. Subsequent research showed it to also predict mortality and specific conditions^24^.

Directly measuring individual-level functioning in this way addresses many of the limitations of previous research into health trends. It provides a well-validated continuous outcome relevant to both robust and less robust adults, allows consideration of patterns and trends both in intrinsic capacity and in each of its subdomains, and takes into account the complex correlations between the many characteristics that underpin this structure.

Taking advantage of this approach, we undertook a longitudinal analysis of cohort trends in intrinsic capacity to determine whether older adults in England and China are experiencing the same, better or worse capacity than people of similar ages in the past. Building on our previous analysis, we used confirmatory factor analysis (CFA) of ELSA data to measure intrinsic capacity and subdomains of capacity across multiple waves, and then used multilevel growth curve models to examine the trends between different birth cohorts. We then applied the same methods for a comparative analysis of the CHARLS cohort. We limited our analysis to variables that were directly comparable between the two studies.

This complex analytical approach differs from most previous work in this field and offers advantages beyond simply using the intrinsic capacity construct as the study outcome. In particular, it allows us to distinguish between trends in the level of capacity with which participants enter older ages and trends in the rate of decline they subsequently experience. This enables a more nuanced consideration of the possible causes of any observed differences between birth cohorts.

## Results

### Main analyses: ELSA

The sample for the main analyses (ELSA) included 14,710 participants aged 60 and older, including 53.3% women (*n* = 7,841). The median birth year was 1940 (interquartile range (IQR) = 1931–1948); the median number of observations was four (IQR = 2–6).

**Fig. 1:**
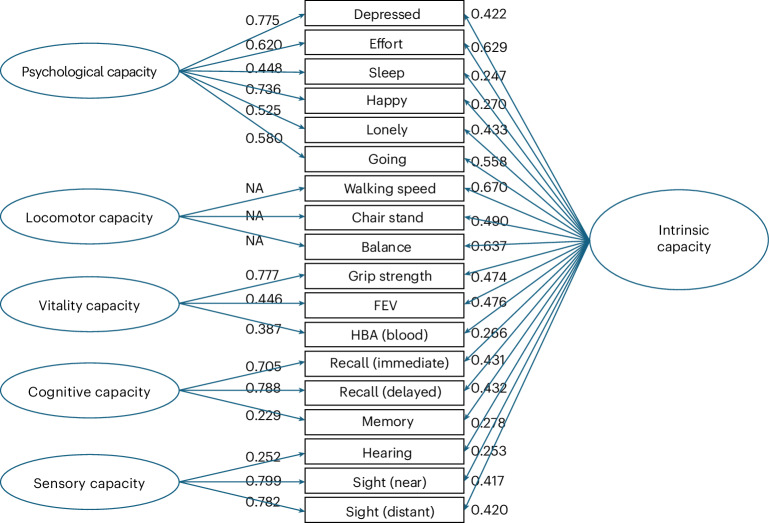
Factor loadings for ELSA. A total of 14,710 participants from the ELSA waves 1–9 (2002–2019) were used for the CFA. Standardized loadings were derived from the scalar bifactor models for intrinsic capacity and from the correlated factor models for each of the subdomains. NA, not applicable.

The results of the bifactor CFA model are shown in Fig. 1; details of the measures used are available in the Supplementary Information. Models with a bifactor structure consisting of intrinsic capacity and five capacity subdomains (cognitive, locomotor, sensory, psychological and vitality, as in ref. ^9^) led to nonsignificant loadings of the locomotor indicators on their subdomain, suggesting the collapse of this specific factor once the common factor (intrinsic capacity) had accounted for common variance across these indicators. Therefore, the bifactor model was respecified, removing the ‘locomotor’ subdomain. The updated configural bifactor model in ELSA showed good fit (Table 1). Additional equality constraints to the loadings and thresholds across time points did not lead to a loss in fit; rather, fit increased because of increased model parsimony. Based on these results, scalar invariance of the proposed intrinsic capacity bifactor model was assumed to hold; similar evidence was found for the correlated factor model, thus enabling comparisons of the levels in intrinsic capacity factors and all the different subdomains over time.

**Table 1:** Results from the measurement invariance testing

	Model	Invariance level	Chi-squared (d.f.)	RMSEA (90% CI)	CFI	TLI	ΔRMSEA	ΔCFI
Main analysis (ELSA), *n* = 3,246	Bifactor model	Configural	1,691 (357)	0.034 (0.032 to 0.036)	0.981	0.975	NA	NA
		Scalar	1,962 (441)	0.033 (0.031 to 0.034)	0.978	0.977	0.001	−0.003
	Correlated factors model	Configural	1,874 (375)	0.035 (0.034 to 0.037)	0.978	0.973	NA	NA
		Scalar	2,374 (429)	0.037 (0.036 to 0.039)	0.972	0.970	−0.002	−0.006
Comparative analysis (CHARLS), *n* = 5,571	Bifactor model	Configural	2,378 (234)	0.041 (0.039 to 0.042)	0.958	0.945	NA	NA
		Scalar	2,789 (289)	0.039 (0.038 to 0.041)	0.951	0.948	0.002	−0.007
	Correlated factors model	Configural	2,118 (253)	0.036 (0.035 to 0.038)	0.963	0.956	NA	NA
		Scalar	2,967 (289)	0.041 (0.039 to 0.042)	0.947	0.944	−0.005	−0.016

Scalar invariance is usually deemed to hold if the difference between the configural model in the RMSEA (ΔRMSEA) and the comparative fit index (CFI) is smaller than 0.015 and 0.010, respectively. Configural invariance is usually deemed to hold if the values of the RMSEA and Tucker–Lewis index (TLI) for the configural model are below 0.060 and above 0.950, respectively^24^. ΔCFI, difference in TLI; NA, not applicable.

**Fig. 2:**
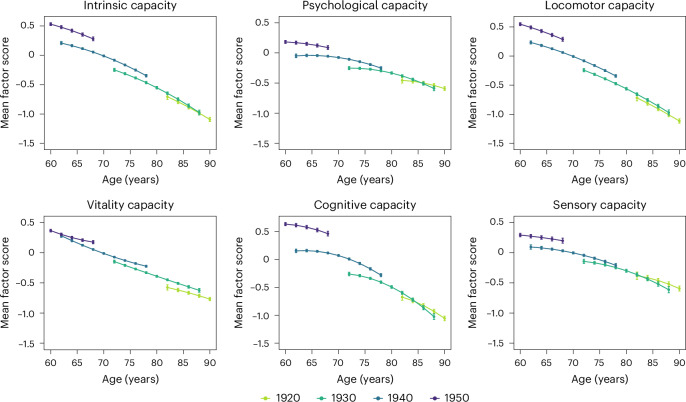
Intrinsic capacity and subdomain mean factor scores according to birth cohort and age in ELSA (main analyses). The plots depict the point estimates and 95% CIs of the marginal mean predicted levels from the multilevel growth curve models for 14,710 participants from the ELSA waves 1–9 (2002–2019). Predicted levels were derived from bifactor models for intrinsic capacity and from correlated factor models for each of the subdomains.

Factor scores were then derived from the bifactor model (intrinsic capacity) and correlated factor models (psychological, locomotor, vitality, cognitive and sensory capacities), including all observations with at least partial information between waves 1 and 9 of ELSA. The results of the multilevel growth curve models computed using these scores are shown in Table 2; the corresponding marginal mean predicted levels for the different birth cohorts are plotted according to age in Fig. 2. All confidence intervals (CIs) were constructed at a 95% confidence level.

**Table 2:** Results from the multilevel growth curve models performed in ELSA and CHARLS

		Main analyses (ELSA)			Comparative analyses (CHARLS)		
		Coefficient	95% CI	*P*	Coefficient	95% CI	*P*
Intrinsic capacity	Linear change	−0.041	−0.055 to −0.027	<0.001	−0.033	−0.044 to −0.022	<0.001
	Quadratic change	−0.001	−0.002 to 0	0.031	NA	NA	NA
	Birth year	0.046	0.043 to 0.048	<0.001	0.035	0.032 to 0.037	<0.001
	Linear change birth year interaction	0.001	0 to 0.002	0.004	0.002	0.001 to 0.002	<0.001
	Quadratic change birth year interaction	0	0.000	0.945	NA	NA	NA
	Intercept	−0.706	−0.753 to −0.659	<0.001	−0.518	−0.551 to −0.484	<0.001
	Slope variance	0.001	0.001 to 0.001	NA	0.037	0.035 to 0.039	NA
	Intercept variance	0.397	0.376 to 0.419	NA	0.572	0.551 to 0.593	NA
	Slope-intercept covariance	−0.010	−0.011 to −0.008	NA	−0.073	−0.079 to −0.068	NA
Psychological capacity	Linear change	*−*0.004	*−*0.017 to 0.008	0.491	0.026	0.014 to 0.039	<0.001
	Quadratic change	*−*0.002	*−*0.002 to −0.001	<0.001	NA	NA	NA
	Birth year	0.020	0.018 to 0.023	<0.001	0.014	0.012 to 0.017	<0.001
	Linear change birth year interaction	0.001	0 to 0.001	0.135	*−*0.001	*−*0.002 to 0	0.013
	Quadratic change birth year interaction	0	0 to 0	0.286	NA	NA	NA
	Intercept	*−*0.455	*−*0.500 to −0.411	<0.001	*−*0.269	*−*0.304 to −0.234	<0.001
	Slope variance	0.001	0.001 to 0.001	NA	0.061	0.058 to 0.064	NA
	Intercept variance	0.355	0.336 to 0.374	NA	0.683	0.659 to 0.709	NA
	Slope-intercept covariance	−0.009	−0.011 to −0.008	NA	−0.119	−0.126 to −0.112	NA
Locomotor capacity	Linear change	*−*0.044	*−*0.060 to −0.029	<0.001	*−*0.020	*−*0.032 to −0.007	0.003
	Quadratic change	*−*0.001	*−*0.002 to 0	0.175	NA	NA	NA
	Birth year	0.048	0.045 to 0.051	<0.001	0.034	0.032 to 0.036	<0.001
	Linear change birth year interaction	0.001	0 to 0.002	0.011	0.003	0.003 to 0.004	<0.001
	Quadratic change birth year interaction	0	0 to 0	0.891	NA	NA	NA
	Intercept	*−*0.719	*−*0.770 to −0.668	<0.001	*−*0.597	*−*0.632 to −0.562	<0.001
	Slope variance	0.001	0.001 to 0.001	NA	0.045	0.043 to 0.048	NA
	Intercept variance	0.420	0.396 to 0.446	NA	0.529	0.506 to 0.552	NA
	Slope-intercept covariance	−0.011	−0.013 to −0.009	NA	−0.093	−0.100 to −0.087	NA
Vitality capacity	Linear change	*−*0.020	*−*0.032 to −0.008	0.001	*−*0.065	*−*0.079 to −0.052	<0.001
	Quadratic change	0	*−*0.001 to 0	0.196	NA	NA	NA
	Birth year	0.043	0.040 to 0.045	<0.001	0.037	0.034 to 0.039	<0.001
	Linear change birth year interaction	*−*0.001	*−*0.002 to 0	0.001	0.003	0.002 to 0.003	<0.001
	Quadratic change birth year interaction	0	0 to 0	0.002	NA	NA	NA
	Intercept	*−*0.576	*−*0.619 to −0.533	<0.001	*−*0.498	*−*0.533 to −0.463	<0.001
	Slope variance	0	0 to 0	NA	NA	NA	NA
	Intercept variance	0.280	0.265 to 0.296	NA	0.409	0.398 to 0.421	NA
	Slope-intercept covariance	−0.006	−0.007 to −0.005	NA	NA	NA	NA
Cognitive capacity	Linear change	*−*0.027	*−*0.044 to −0.011	0.001	*−*0.089	*−*0.102 to −0.077	<0.001
	Quadratic change	*−*0.003	*−*0.004 to −0.002	<0.001	NA	NA	NA
	Birth year	0.042	0.038 to 0.045	<0.001	0.032	0.030 to 0.035	<0.001
	Linear change birth year interaction	0.002	0.001 to 0.003	<0.001	0.002	0.001 to 0.003	<0.001
	Quadratic change birth year interaction	0	0 to 0	0.316	NA	NA	NA
	Intercept	*−*0.673	*−*0.730 to −0.616	<0.001	*−*0.370	*−*0.404 to −0.335	<0.001
	Slope variance	0.002	0.001 to 0.002	NA	0.062	0.059 to 0.065	NA
	Intercept variance	0.388	0.366 to 0.413	NA	0.683	0.654 to 0.714	NA
	Slope-intercept covariance	−0.008	−0.010 to −0.006	NA	−0.116	−0.125 to −0.108	NA
Sensory capacity	Linear change	*−*0.013	*−*0.031 to 0.004	0.139	0.015	0.002 to 0.029	0.025
	Quadratic change	*−*0.002	*−*0.003 to −0.001	0.003	NA	NA	NA
	Birth year	0.024	0.020 to 0.027	<0.001	0.022	0.019 to 0.025	<0.001
	Linear change birth year interaction	0	*−*0.001 to 0.001	0.412	*−*0.001	*−*0.002 to −0.001	0.001
	Quadratic change birth year interaction	0	0 to 0	0.119	NA	NA	NA
	Intercept	*−*0.382	*−*0.443 to −0.320	<0.001	*−*0.298	*−*0.336 to −0.260	<0.001
	Slope variance	0.002	0.002 to 0.002	NA	0.062	0.059 to 0.066	NA
	Intercept variance	0.489	0.462 to 0.518	NA	0.719	0.691 to 0.748	NA
	Slope-intercept covariance	−0.018	−0.021 to −0.016	NA	−0.133	−0.142 to −0.125	

A total of 14,710 participants from the ELSA waves 1–9 (2002–2019) and 11,411 participants from the CHARLS waves 1–3 (2011–2015) were used for the multilevel growth curve models. Coefficients and 95% CIs were derived from the bifactor models for intrinsic capacity and from the correlated factor models for each of the subdomains. Two-sided *P* values (unadjusted for multiple comparisons) were obtained, with *P* values below 0.001 presented as *P* < 0.001. Longitudinal weights were used to restore representativeness to participants aged 50 and older and living in England; survey weights were used to restore representativeness to participants aged 45 and older and living in China. NA, not applicable.

More recent cohorts entered older ages with significantly higher levels of intrinsic capacity (birth year = 0.046, 95% CI = 0.043 to 0.048, *P* < 0.001). While intrinsic capacity levels declined with increasing age across all cohorts, these declines were less steep for more recent cohorts than for earlier ones (linear change * birth year = 0.001, 95% CI = 0 to 0.002, *P* = 0.004).

More recent cohorts also entered older ages with significantly higher levels for each subdomain of capacity, these improvements being largest in the locomotor, vitality and cognitive capacity subdomains. As with intrinsic capacity as a whole, declines with increasing age were observed across all subdomains. However, declines in the locomotor and cognitive subdomains were less steep among more recent cohorts. For vitality capacity, they were initially faster among younger cohorts (linear change * birth year = −0.001, 95% CI = −0.002 to 0, *P* = 0.001) but subsequently followed more stable levels over time (quadratic change * birth year = 0, 95% CI = 0 to 0, *P* = 0.002).

To quantify the observed improvements shown in Fig. 2, we calculated the marginal mean predicted levels for each age according to cohort (Table 3 and Extended Data Tables 2–4). Even when comparisons were limited to cohort participants for whom data are available at the same age, the observed improvements were large. For example, the intrinsic capacity of the cohort born in 1950 at age 68 was 0.280 (95% CI = 0.248 to 0.313), significantly higher than the 0.208 (95% CI = 0.183 to 0.233) of the cohort born in 1940 at age 62. The pattern was similar for psychological, locomotor, cognitive and sensory capacities, although the improvement was most pronounced for cognitive capacity. The greatest improvements were between the most recent cohort (1950) and the 1940 cohort, although direct comparisons between earlier cohorts also showed significant improvements in the more recent cohort (Extended Data Tables 2–4). If these directly observed trends were extrapolated to compare the earliest with the most recent cohort, the improvements would be significantly greater than those we could observe directly.

**Table 3:** Scores according to birth cohort and age in ELSA (1940 and 1950 cohorts)

Intrinsic capacity							Locomotor capacity					
	1950s cohort			1940s cohort			1950s cohort			1940s cohort		
Age	Mean	Lower	Higher	Mean	Lower	Higher	Mean	Lower	Higher	Mean	Lower	Higher
60	0.531	0.506	0.555				0.548	0.522	0.573			
62	0.479	0.454	0.504	0.208	0.183	0.233	0.492	0.466	0.517	0.233	0.208	0.259
64	0.42	0.394	0.446	0.164	0.145	0.183	0.43	0.404	0.455	0.182	0.163	0.201
66	0.354	0.327	0.381	0.113	0.098	0.129	0.361	0.334	0.389	0.125	0.109	0.141
68	0.280	0.248	0.313	0.055	0.04	0.07	0.287	0.254	0.32	0.062	0.047	0.077
70				−0.011	−0.026	0.005				−0.007	−0.023	0.009
72				−0.084	−0.099	−0.068				−0.082	−0.098	−0.066
74				−0.164	−0.18	−0.148				−0.163	−0.179	−0.147
76				−0.252	−0.269	−0.235				−0.249	−0.267	−0.231
78				−0.347	−0.368	−0.326				−0.342	−0.364	−0.319

	Vitality capacity						Cognitive capacity					
Age	1950s cohort			1940s cohort			1950s cohort			1940s cohort		
60	0.365	0.344	0.386				0.638	0.61	0.666			
62	0.303	0.282	0.324	0.280	0.255	0.306	0.618	0.588	0.647	0.157	0.126	0.189
64	0.251	0.23	0.272	0.2	0.181	0.218	0.582	0.551	0.613	0.162	0.14	0.183
66	0.208	0.186	0.23	0.124	0.109	0.138	0.532	0.498	0.566	0.149	0.132	0.166
68	0.174	0.148	0.201	0.053	0.04	0.066	0.467	0.425	0.508	0.12	0.103	0.136
70				−0.013	−0.025	0				0.074	0.056	0.091
72				−0.073	−0.086	−0.061				0.011	−0.007	0.029
74				−0.129	−0.141	−0.117				−0.069	−0.088	−0.05
76				−0.179	−0.193	−0.166				−0.165	−0.187	−0.144
78				−0.225	−0.242	−0.208				−0.278	−0.306	−0.251

	Psychological capacity						Sensory capacity					
Age	1950s cohort			1940s cohort			1950s cohort			1940s cohort		
60	0.181	0.157	0.206				0.292	0.264	0.32			
62	0.169	0.144	0.195	−0.05	−0.079	−0.02	0.274	0.245	0.303	0.094	0.058	0.13
64	0.15	0.125	0.175	−0.042	−0.063	−0.022	0.252	0.223	0.281	0.08	0.055	0.104
66	0.123	0.096	0.149	−0.044	−0.061	−0.028	0.227	0.195	0.259	0.059	0.04	0.077
68	0.088	0.056	0.119	−0.056	−0.071	−0.041	0.199	0.157	0.24	0.031	0.014	0.048
70				−0.076	−0.092	−0.061				−0.004	−0.021	0.014
72				−0.106	−0.122	−0.091				−0.045	−0.063	−0.027
74				−0.145	−0.161	−0.13				−0.092	−0.111	−0.074
76				−0.194	−0.21	−0.177				−0.147	−0.167	−0.126
78				−0.251	−0.272	−0.231				−0.208	−0.235	−0.18

Table 3 reports the marginal mean scores of intrinsic capacity for the 1940 and 1950 birth cohorts with 95% CIs (lower and upper bounds). Blank cells indicate not applicable.

### Comparative analyses: CHARLS

The sample for the comparative analyses (CHARLS) included 11,411 participants aged 60 and older, including 50.0% women (*n* = 5,706). The median birth year was 1947 (IQR = 1941–1951); the median number of observations was two (IQR = 1–3).

**Fig. 3:**
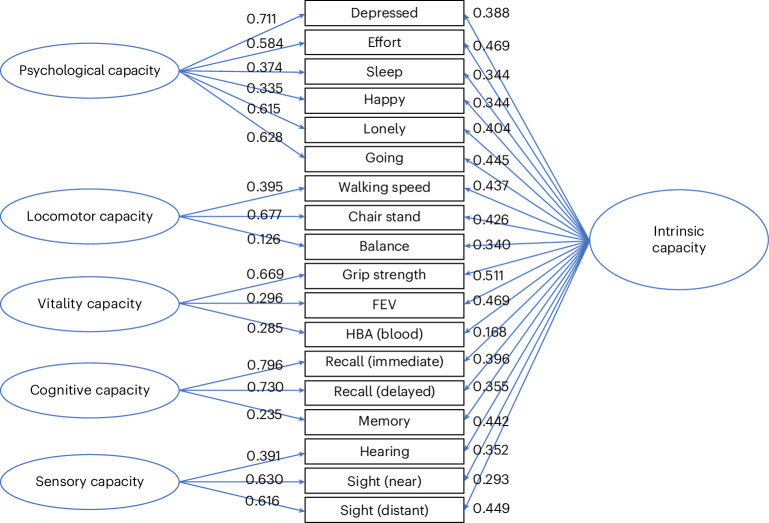
Factor loadings for CHARLS. A total of 11,411 participants from the CHARLS waves 1–3 (2011–2015) were used for the CFA. Standardized loadings were derived from the scalar bifactor models for intrinsic capacity and from the correlated factor models for each of the subdomains.

The results of the CFA modeling of the CHARLS data are shown in Fig. 3. Measurement invariance testing (Table 1, lower section) suggested that scalar invariance held for the bifactor model (intrinsic capacity). However, the restrictions imposed to fit the scalar model in the correlated factor model resulted in a substantial loss in fit according to the change in CFI (the change in root mean square error of approximation (RMSEA) was within the boundaries). Factor scores for each subdomain were derived but interpreted with additional caution in the case of the subdomains, as changes in score levels could be due to differences in the measurement parameters across the time points. Therefore, trajectories in the subdomains for the CHARLS need to be considered with caution.

**Fig. 4:**
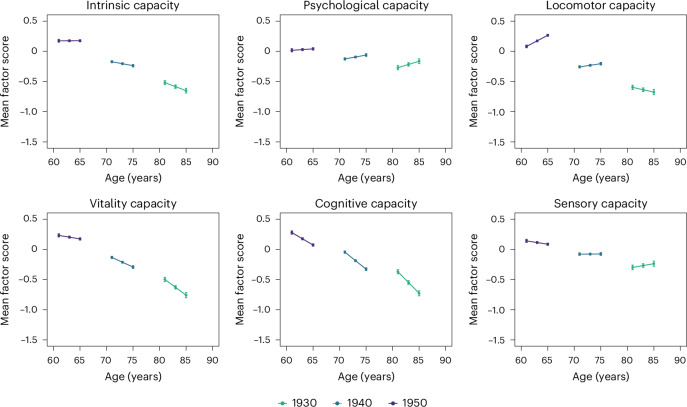
Intrinsic capacity and subdomain mean factor scores according to birth cohort and age in CHARLS (comparative analyses). The plots depict the point estimates and 95% CIs of the marginal mean predicted levels from the multilevel growth curve models for 11,411 participants from the CHARLS waves 1–3 (2011–2015). Predicted levels were derived from bifactor models for intrinsic capacity and from correlated factor models for each of the subdomains.

Coefficients from the multilevel growth curve models estimated with the CHARLS data as part of the comparative analyses are shown in Table 2 (right); the corresponding marginal mean predicted levels for the different birth years are plotted according to age in Fig. 4. Consistent with the main analyses for ELSA, more recent cohorts entered older ages with higher levels of capacity (birth year = 0.035, 95% CI = 0.032 to 0.037, *P* < 0.001). The largest improvements were found for the vitality capacity subdomain, followed by locomotor, cognitive, sensory and, finally, psychological factors. Intrinsic capacity declined significantly with age; subsequent declines for more recent cohorts were less steep than for earlier cohorts (linear change * birth year = 0.002, 95% CI = 0.001 to 0.002, *P* < 0.001). The findings for the subdomains may be at least partly attributed to the lack of measurement invariance of the subdomain analysis outlined above. As with the main analysis, steeper declines with increasing age were observed among earlier cohorts in the locomotor, vitality and cognitive capacity subdomains. However, for the psychological and sensory subdomains, changes over time were positive with increasing age overall (linear change), with more recent cohorts experiencing smaller improvements (linear change * birth year).

### Sensitivity analyses

**Extended Data Fig. 1:**
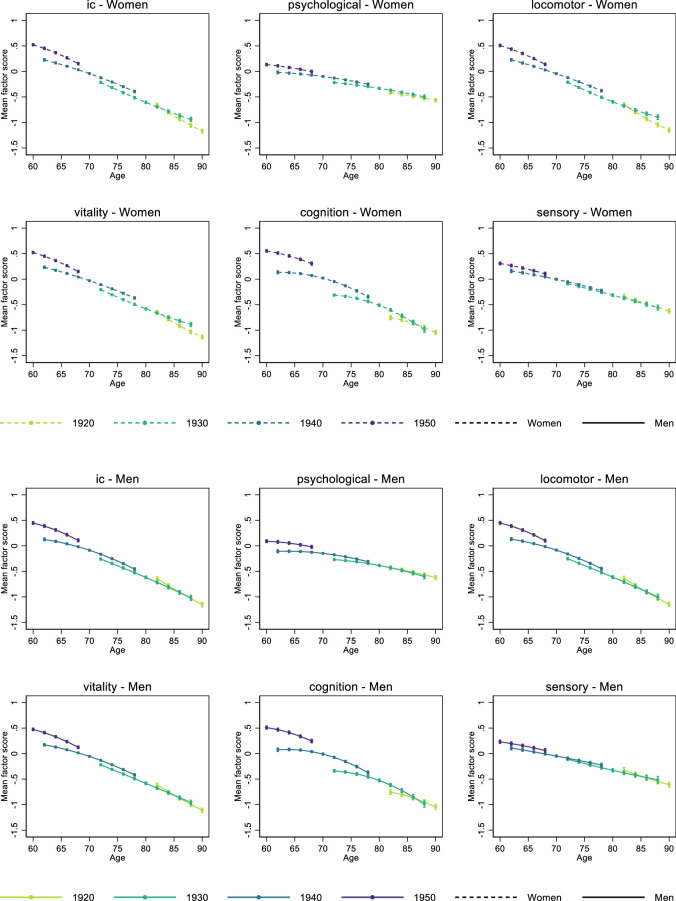
Intrinsic capacity and subdomains mean factor scores by birth cohort, age, and gender in ELSA. Note: Plots depict the point estimates and 95% confidence intervals of the marginal mean predicted levels from the multilevel growth curve models for 7,841 different women and 6,809 different men from the English Longitudinal Study on Ageing (ELSA) waves 1 (2002) to 9 (2019). Predicted levels were derived from bifactor models for intrinsic capacity and from the correlated factor models for each of the subdomains.

**Extended Data Fig. 2:**
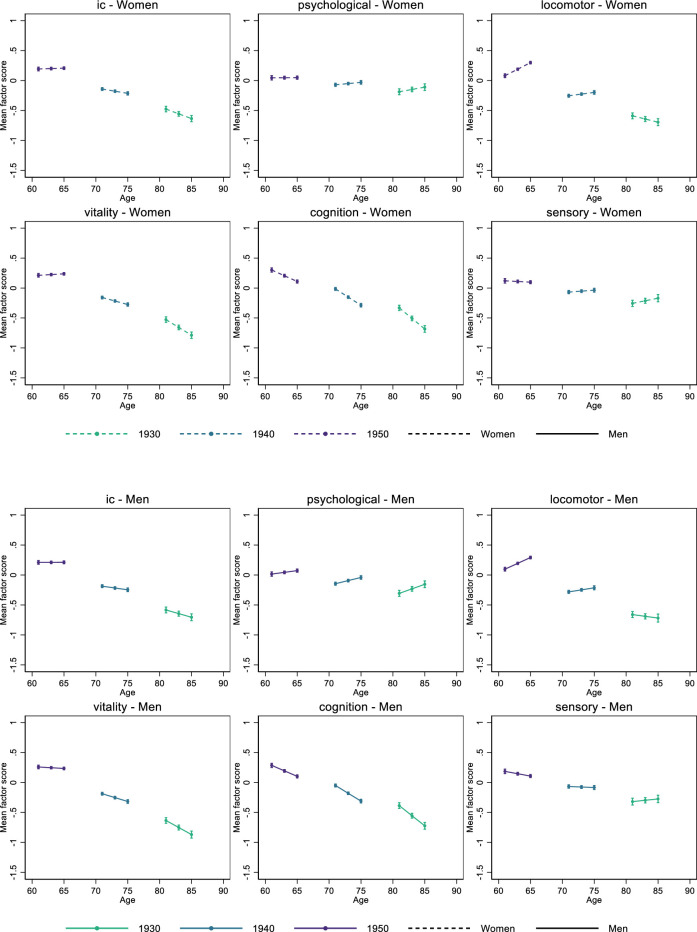
Stratified analysis by gender in CHARLS. Note: Plots depict the point estimates and 95% confidence intervals of the marginal mean predicted levels from the multilevel growth curve models for 5,706 different women and 5,705 different men from the China Health and Retirement Longitudinal Study waves 1 (2011) to 3 (2015). Predicted levels were derived from bifactor models for intrinsic capacity and from the correlated factor models for each of the subdomains.

We performed sensitivity analyses in ELSA, replacing the longitudinal weights with cross-sectional survey weights and found similar results. We also undertook an additional analysis to determine whether the observed trajectories varied according to sex (Supplementary Information). Measurement invariance did not hold across both waves and sexes in either ELSA or CHARLS. However, measurement invariance was held over time in the sexes in most cases except for the correlated factor model in CHARLS (Extended Data Table 5). Therefore, factor scores were derived separately for each sex and multilevel growth curve models were estimated according to sex using the corresponding factor scores. Although direct comparisons across the sexes could not be made as they would be biased by differences in the measurement of the intrinsic capacity factor and its subdomains, within-sex trajectories mirrored those found in the overall analyses (Extended Data Tables 6 and 7 and Extended Data Figs. 1 and 2).

## Discussion

Our research suggests that there have been significant improvements in functioning in more recent cohorts of older people in both England and China. Within ELSA, more recent cohorts entered older ages with higher levels of intrinsic capacity; subsequent declines were less steep than for earlier cohorts. Improvements were seen in all subdomains. Trajectories were similar for men and women and largely consistent across both countries, although our analysis was limited by the lesser availability of wave data in CHARLS.

The observed improvements are substantial. To avoid undue extrapolation, we limited our assessment to direct comparisons of capacity in participants of different cohorts at the same age. Currently, the overlap between adjacent cohorts in the ELSA study is 6 years; participants of nonadjacent cohorts cannot be directly compared. However, even with these limitations, we still found that a 68-year-old ELSA participant born in 1950 had higher intrinsic capacity than a 62-year-old born just 10 years earlier. Improvement in cognition was even more substantial. When comparing earlier cohorts, additional improvements were observed, although the gains between these cohorts are not quite as large as between the 1940 and 1950 cohorts. Thus, while our models suggest that today’s 70-year-olds have the equivalent functioning to substantially younger adults in previous generations (perhaps 70 really is the new 60), our direct assessments can only confirm that in this study 68 is the new 62.

Most previous research directly examining functional trends has been limited to studies of severe disability, and the findings have been inconsistent. For example, in the UK, a comparison of similar cohorts of people over the age of 65 between 1991 and 2011 suggested that only 36.4% of the extra years of life gained for men and 4.8% for women were experienced with no level of care dependency^12^. On the other hand, analysis of ELSA data from 2002 to 2016 found that ADL limitations declined in those aged 55–64 years^13^. A recent study in ELSA of trends in a frailty index, including a broad range of health indices such as ADLs and IADLs, similarly found improvements in more recent cohorts, although these were inequitably spread and may be waning^20^. In China, some studies suggested that the age-adjusted prevalence of ADL loss may be declining^15^, while others found that limitations in ADLs and IADLs may be increasing^16^ or that there may be a V-shaped trend for ADLs^14^.

These inconsistencies are likely to arise partly from the wide variety of measures used^25^. They may also be influenced by changing patterns of institutionalization. Some measures (particularly IADLs) also have difficulty distinguishing between changes that may be occurring in the individual and those that might result from changes in the environment. For example, a common IADL question relates to how easy it is to use a phone, yet phone type and use have changed with time^26^.

However, the most fundamental limitation of studies of severe disability is that they tell us little about the functioning of people in relatively robust health. In contrast to previous research, the continuous nature of the intrinsic capacity construct and its subdomains allowed us to examine milder and earlier forms of age-related limitations than previous analyses, and to consider individual-level changes independent of any changes that may have occurred in contextual factors.

The improvements in functioning we identified have no obvious single driver. However, our innovative approach allows us to distinguish between two distinct mechanisms through which determinants may operate: influencing the level of capacity with which one enters older ages or influencing subsequent declines in capacity (note that a single determinant may affect both of these). The peak level of capacity with which an individual enters older ages will necessarily be determined by factors operating across their earlier lives. Age-related declines might be influenced by both earlier life factors (for example, intrauterine nutrition and smoking behaviors) and later life influences (for example, medical treatments).

In our analysis, most of the improvements in functioning could be attributed to more recent birth cohorts entering older ages with higher levels of capacity rather than the lesser influence of slower late-life declines. This finding is consistent with a strong influence of early life factors and higher early life functional peaks. These trends are starkest for cognitive capacity and align with research suggesting that greater educational opportunities in childhood may be one explanation for the 13% per-decade decline in the incidence rate of dementia observed in Europe and North America over the past 25 years^27,28^. However, we observed similar trends for multiple subdomains of capacity, suggesting that the direct impact of education alone is unlikely to fully explain these improvements. Other early life influences, particularly socioeconomic status and better nutrition (of both mothers and children), may therefore also have played a role. Our previous research in CHARLS found that early life factors such as these directly explained around 14% of the intrinsic capacity inequalities observed in older adults in China and a further 29% of these inequalities through their influence on current socioeconomic status^29^. Even the antenatal experience of mothers may influence the risk of chronic conditions at older ages in their children^30,31^.

Changing exposures to infectious diseases may also have been important. One hypothesized driver of the multiple chronic conditions associated with increasing age is immunosenescence, which is influenced by multiple factors, including infections and nutrition^32^. Changes in exposure to common pathogens across the life course related to better sanitation and other environmental factors could thus have influenced underlying age-associated biology.

In examining the possible role of these broad social influences, it is also important to consider period effects. The cohorts included in these studies were born between 1920 and 1959 for ELSA and 1930 and 1955 for CHARLS; early life experiences from these periods, which include the Great Depression, World War II and the Chinese Civil War, have probably been an important influence^33^.

Therefore, it is possible that improvements to education, nutrition and sanitation across the twentieth century, punctuated by periodic societal upheavals, might explain many of the trends we observed. If this occurred through slowing the complex and dynamic processes that drive aging, it might also be expected to reduce the risk of chronic diseases and be associated with a fall in the incidence of these conditions. However, this is difficult to confirm because, for many reasons, incidence trends are challenging to estimate^34,35^; disease incidence might also be influenced by other factors, such as improved medical care for biological risk factors. The studies that have been done document falling incidence in the UK for some conditions (for example, cardiovascular disease^36^ and osteoarthritis^37^), but not others (for example, certain cancers). In China, the incidence of stroke seems to be declining, while the incidence of other chronic conditions appears to be increasing^38^.

Another key factor in the slower rates of decline we observed is likely to be greater access to healthcare or improved medical treatments. In general, these would operate by reducing the functional consequences of specific conditions rather than influencing the underlying process of aging. They also remain inequitably spread^39^. Detection and management of biological risk factors may have improved, reducing their impact (and potentially increasing their prevalence), although observed trends are inconsistent. In ELSA, rates of awareness of hypertension, treatment of hypertension and the proportion of treated participants who achieved recommended targets have increased over time^40^. Management of hypertension in China has also improved, although the age-standardized prevalence of high blood pressure has increased significantly^41^. A reported increase in the prevalence of diagnosed diabetes in ELSA participants from 7.7% in 2004 to 11.5% in 2012 would also be consistent with better detection and possibly management^42^. However, between 2004 and 2012, there was also a significant rise in the prevalence of undiagnosed diabetes and only a very small decrease in the proportion of participants with diabetes who were unaware of this condition^42^.

A further influence may have been changes in health behaviors. However, behaviors and risk factors in the UK and China have trended in multiple directions over the past 25 years. Age-standardized prevalence estimates suggest that between 1990 (or 2000, depending on data availability) and 2015, tobacco use fell in the UK but remained relatively steady in China, while the prevalence of being overweight rose in both countries^43^. Trends in physical activity in the UK and elsewhere are hard to determine, but may have declined over time^44,45^. In China, physical activity from work and domestic activities may have fallen by around 50% between 1991 and 2011 (ref. ^46^), although other analyses suggest a more stable long-term trend in China, at least from 2000 to 2015 (ref. ^47^).

In summary, the explanations for the improvements we have observed are probably complex and relate to social change across most of the past century, as well as both medical and public health advances.

Our analysis has many strengths, including the representative nature of the samples. The instruments underpinning our measure are widely used and, where possible, objective. They distinguish between individual-level change and changes that might have occurred in the physical and social environments the individual inhabits.

However, when considering these findings, it is important to understand the limitations of our research. We explored the typical experience of cohorts, and this probably masks significant intracohort heterogeneity. This has already been reported in ELSA^20^. We considered intracohort heterogeneity in our sex-based analysis, which suggested that the improvements we observed were not limited to one sex. However, research in other cohorts suggests that any positive health trends are probably greater for more advantaged socioeconomic groups, and we cannot exclude this possibility^48,49^. Thus, while 70 may well be the new 60 for some, for many others it may still be the same 70 (or worse). The trends we are observing at the population level are the aggregate of many individual trajectories.

Nor should the findings reported in this article be extrapolated to other countries or settings since they describe trends in two specific settings over specific periods of time. As discussed above, several period effects probably influenced our findings. The dramatic improvements for participants born around 1950 compared to those born around 1940 are probably significantly influenced by World War II. In turn, both of these birth cohorts showed improvements over the 1930 birth cohort, which experienced the Great Depression. People born after these major geopolitical events may have had very different experiences. Indeed, evidence from multiple countries indicates that the trend of increasing longevity may be slowing, and risk factor patterns may be worsening among people currently in midlife^50^. Both trends will probably affect future health expectancy^51^.

Changing patterns of institutionalization may have influenced our findings as both studies use community-based samples. In the UK, the number of nursing home admissions for those aged 65 and older fell by 18% between 2014 and 2022 (ref. ^52^). However, this shift in institutionalization in England would operate against the positive trends we observed, while in China, institutionalization rates remain low at around 1% and recent emphasis has been on community-based care services^53^.

It is also probable that participants with worse intrinsic capacity were disproportionately excluded from the study samples, particularly for older ages and cohorts. However, any resulting survivor bias would probably be greater for older cohorts and any effect would be to underestimate the positive trends we observed.

Because of the complexity of the measurement models, we could not embed the latent variables themselves in the analyses of the longitudinal trajectories. Rather, we derived factor scores and analyzed these over time. These factor scores are assumed to be free of error (as would be any other observed outcome), so it is important to acknowledge that measurement error may still be a source of bias in this study.

It is also possible that self-report effects may be at play in the psychological and sensory subdomains, while trends in the sensory domain may have been affected by changes in access to hearing and visual supports. However, the steepest improvements in capacity were found in subdomains measured with objective indicators, suggesting that they are not explained by reporting bias. Finally, attrition in the two studies also needs to be considered as a possible influence on our findings. However, sample attrition in ELSA was previously shown not to significantly affect estimates of disease prevalence, suggesting that any influence is probably minor^54^.

Our findings suggest several avenues for further research. If they can be replicated and the limitations addressed, future studies could examine whether trends vary between settings, how trends might be influenced by socioeconomic and other characteristics such as race or ethnicity, and why these trends may be occurring. This might suggest interventions to ensure that the trends we have observed are reinforced and equitably spread.

In the meantime, our analysis strongly suggests that increasing life expectancy in England and China is being accompanied by large increases in health expectancy among more recent cohorts, at least when focusing on people born between 1920 and 1959. This has positive implications for all of us, both as individuals and for society more broadly.

## Methods

This research complies with all relevant ethical regulations. Ethical approval for all the ELSA waves was granted by NHS Research Ethics Committees under the National Research and Ethics Service. The Biomedical Ethics Review Committee of Peking University approved the CHARLS study (IRB00001052-11015). Furthermore, the current study received approval from the University of New South Wales (UNSW) Ethics Committee (no. HC210472).

### Sample characteristics and data collection

ELSA follows a nationally representative sample of the English population aged 50 and older, while CHARLS follows a nationally representative sample of the Chinese population aged 45 and older. Ethical consent has been obtained for all waves and components of ELSA according to the ethical approval system in operation at the time. ELSA wave 11 received ethical approval from the South Central-Berkshire Research Ethics Committee (23/SC/0112). The Biomedical Ethics Review Committee of Peking University approved the CHARLS study (IRB00001052-11015); participants of the CHARLS study provided consent for access to their data for secondary research. Data collection in both cohorts was conducted through face-to-face assessments using computer-assisted personal interviews. In addition, objective measures and blood samples were collected by trained nurses in waves 2, 4 and 6 in ELSA and waves 1 and 3 in CHARLS. The response rates in both ELSA and CHARLS were reasonably high, although they varied across waves. The average follow-up length is about 4.84 years in ELSA and 2.18 years in CHARLS, with attrition rates of 36.3% in ELSA and 45.0% in CHARLS from wave 1 to wave 2. The details regarding the follow-up and missing information are provided in Extended Data Table 1.

We included ELSA and CHARLS participants aged 60 and older with valid information in at least one of the indicators used to measure intrinsic capacity in at least one wave. We focused on participants aged 60 years or more because one of the key measures (walking speed) was only (or mostly) assessed after this age. ELSA currently has nine waves of data available, while five waves are available for CHARLS. Because of the comprehensive measures included in this longitudinal study and the many years of follow-up, we made ELSA (waves 1–9 (2002–2019)) the focus of our main analysis. We then applied the same methods to the CHARLS cohort (waves 1–3 (2011–2015)), but given the shorter follow-up period, we report this as a comparative analysis. More recent data from CHARLS (waves 4 (2018) and 5 (2020)) were not included as, by design, none of the locomotor and vitality capacity subdomain indicators were assessed. This research involved secondary analysis of previously collected data; patients and the public were not involved in any way. No statistical methods were used to predetermine sample sizes but our sample sizes are similar to those reported in previous publications^9,22^.

### Measures

We used data from multiple self-reported and objectively measured tests to create scores for intrinsic capacity and subdomains of capacity consistent with the WHO model of intrinsic capacity^23^. To maximize comparability across the two cohort studies, we focused on the indicators that were present in both ELSA and CHARLS (see the Supplementary Information for details).

Building on our previous analyses, we used a CFA approach to operationalize a set of relevant subdomains^9,22^. These included locomotor capacity (measured using walking speed, chair stand test and balance), cognitive capacity (immediate recall, delayed recall, time orientation/memory), sensory capacity (reported hearing and visual impairments), psychological capacity (affect and sleep as measured using the Center for Epidemiological Studies-Depression Scale^55^ items present in ELSA and CHARLS) and vitality capacity (grip strength, forced expiratory volume (FEV), hemoglobin A (HBA)). Intrinsic capacity was operationalized as a latent common cause of the levels across all indicators (general factor under a bifactor structure) after accounting for the unique shared variance among subsets of indicators as captured by the subdomains.

### Statistical analyses

To ensure that the constructs (that is, the intrinsic capacity general factor and the subdomains) under study were equivalently measured over time, we first used a measurement invariance testing approach^56,57^. In this approach, a multiple-group CFA model without constraints (that is, configural model) was first estimated to assess whether the same factor structure held across time points (that is, configural invariance). Configural invariance was deemed to hold if the values of the RMSEA and the TLI for the configural model were below 0.060 and above 0.950, respectively^58^. Provided that configural invariance held, an additional level of invariance, scalar invariance, was tested, where factor loadings and item intercepts and thresholds were fixed to be equal across time points. Scalar invariance ensures that comparisons of the levels in the constructs are not biased due to differences in the way in which they are measured across time points^56^. As the main aim of this study was to explore the trajectories in those constructs, ensuring that scalar invariance held was crucial. Scalar invariance was deemed to hold if the difference in fit between the scalar and configural models was smaller than 0.015 and 0.010 in the RMSEA and CFI, respectively^59,60^. These models were computed using the data from participants present in all the waves in which all indicators were present (that is, waves 2, 4 and 6).

Once measurement invariance had been tested in waves 2, 4 and 6 in ELSA and waves 1 and 3 in CHARLS, the measurement models were extended to include the remaining waves (waves 1, 3, 5, 7, 8 and 9 in ELSA, wave 2 in CHARLS), where only partial information was available by design. We used weighted least squares mean and variance-adjusted estimation with pairwise deletion to estimate these models and generate factor scores representing the individuals’ levels of intrinsic capacity and each of the subdomains based on the factor models with multiple indicators. The use of pairwise deletion allowed us to obtain estimates of the measurement models in the presence of partial information based on the pattern of relationships between the indicators across the waves, maximizing the use of the information available for each individual^61^. While this approach can provide biased estimates if data are not missing completely at random, it retains more information from the available data, which maximizes the reliability and validity of the model estimates and offers more plausible results compared to other approaches like list-wise deletion^61^. Using alternative estimation procedures with more plausible assumptions (for example, full information maximum likelihood assuming that data are missing at random) was not feasible because of the complexity of the measurement models.

Intrinsic capacity factor scores were derived from the bifactor models, while subdomain factor scores were estimated using correlated factor models where only the subdomains were present and allowed to correlate with each other. We used this approach for the subdomain factor scores because bifactor models would give ‘residualized’ versions of the subdomains, capturing what was left after accounting for the general factor.

Multilevel growth curve models were then used to model change over time in intrinsic capacity and the five capacity subdomains (psychological, locomotor, vitality, cognitive and sensory)^62^. Time was included in the models as the years elapsed since the first wave. Both linear (constant change) and quadratic (accelerated change) terms were included to allow for nonlinear trajectories in ELSA. However, for CHARLS, only linear trajectories were analyzed because of fewer repeated measurements being available. Birth year (in years, centered at 1920 in ELSA and 1930 in CHARLS) was included in the models as a covariate to account for potential differences in the initial levels across cohorts. Interaction terms between birth year and the growth parameters (that is, linear and quadratic) were included in the models to account for potential differences in the rates of change across cohorts. We acknowledged the heterogeneity in the intercepts and rates of change by modeling the random effects of both the intercepts and linear slopes, which were allowed to correlate. Variation in the rates of change over time was captured by the random effects for the linear and constant change. Random effects for the quadratic and accelerated change could not be included because of model estimation and convergence issues.

All models were computed using survey weights to restore representativeness to each study’s population of reference. In the main ELSA analysis, to confirm the robustness of the results to the differential nonresponse to the different waves and to ensure representativeness to participants aged 50 and older, living in England in 2002 and still alive and residing in private households by wave 9, we estimated the models using longitudinal weights^63^. Because these longitudinal weights take into account the differential nonresponse to all the waves, analyses do not rely on the assumption of the data being missing completely at random but rather on them being missing at random after conditioning on the variables used to derive those weights. In CHARLS, survey cross-sectional weights were used for multilevel growth curve models to ensure representativeness to participants aged 50 and older and living in China^64^. All the weights were provided by the ELSA and CHARLS teams. To aid the interpretation of the results, marginal predicted levels were obtained from the models for each of the data collection time points and plotted in the year and age vector plots. Furthermore, these marginal predicted levels were tabulated in age × cohort grids.

### Reporting summary

Further information on research design is available in the Nature Portfolio Reporting Summary linked to this article.

## Supplementary information

Supplementary Information Measurement of intrinsic capacity indicators and additional analyses according to sex.

Reporting Summary

## Figures and Tables

**Extended Data Table 1:**
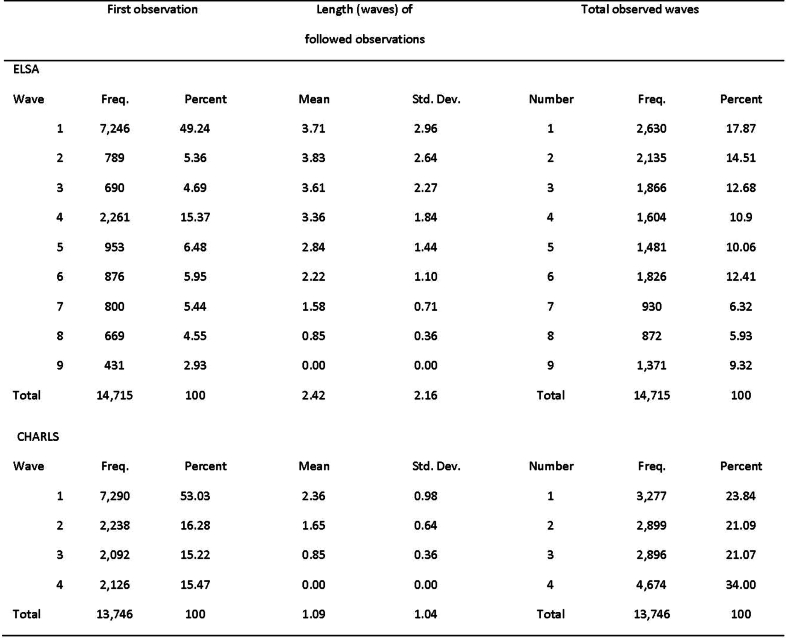
Description of the follow-up information

Each wave represents about 2 years.

**Extended Data Table 2:**
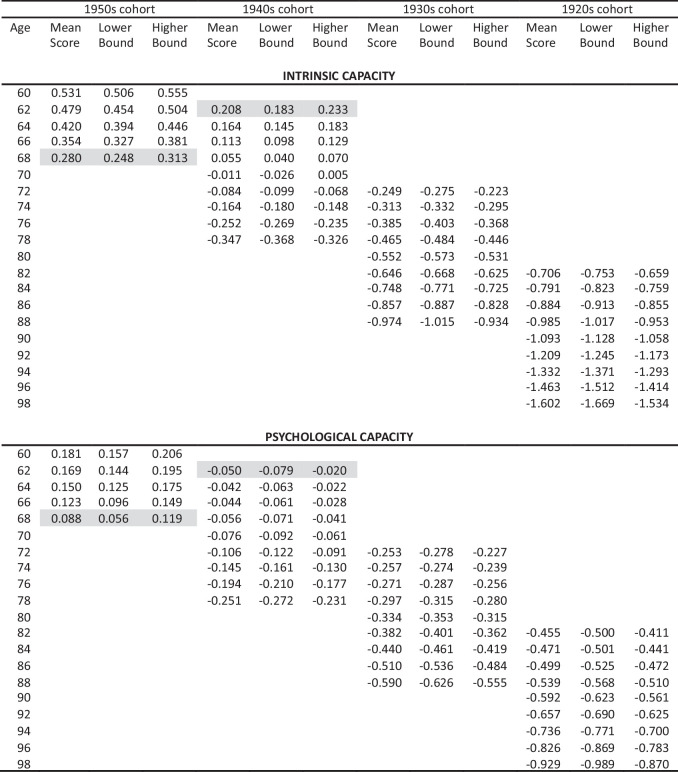
Mean intrinsic capacity and psychological capacity scores according to birth cohort and age in ELSA

The table reports the marginal mean scores of intrinsic capacity for different cohorts with the 95% confidence intervals (lower and higher bounds). The highlights show where capacity at the oldest ages in the 1950 cohort exceeds that for the youngest ages in the 1940 cohort.

**Extended Data Table 3:**
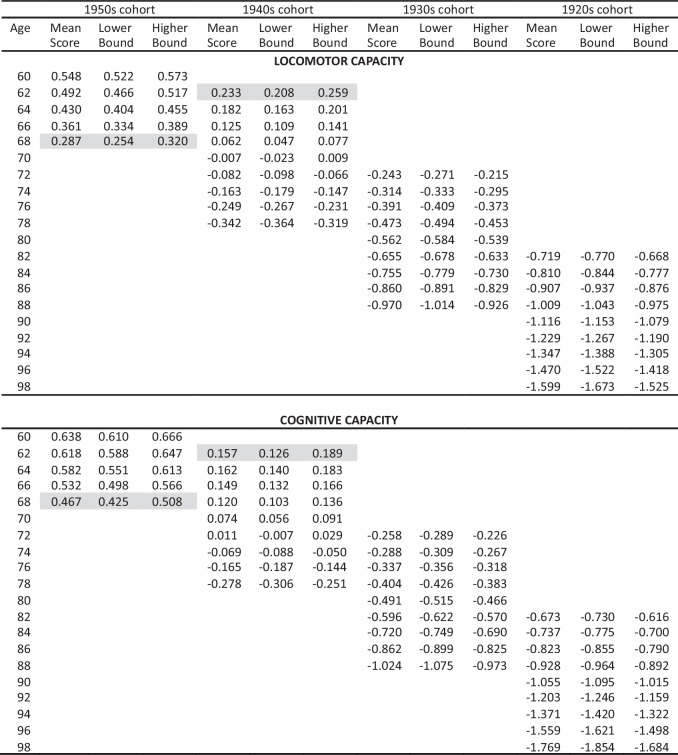
Mean locomotor and cognitive capacity scores according to birth cohort and age in ELSA

The table reports the marginal mean scores of locomotor and cognitive capacity for the different cohorts with the 95% confidence intervals (lower and higher bounds). The highlights show where capacity at the oldest ages in the 1950 cohort exceeds that for the youngest ages in the 1940 cohort.

**Extended Data Table 4:**
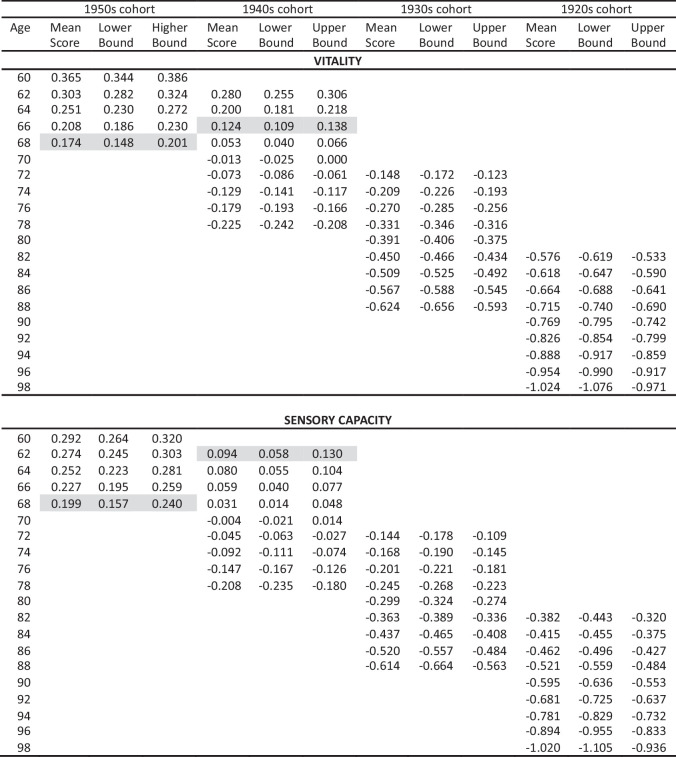
Mean vitality and sensory capacity scores according to birth cohort and age in ELSA

The table reports the marginal mean scores of vitality and sensory capacity for the different cohorts with the 95% confidence intervals (lower and higher bounds). The highlights show where capacity at the oldest ages in the 1950 cohort exceeds that for the youngest ages in the 1940 cohort.

**Extended Data Table 5:**
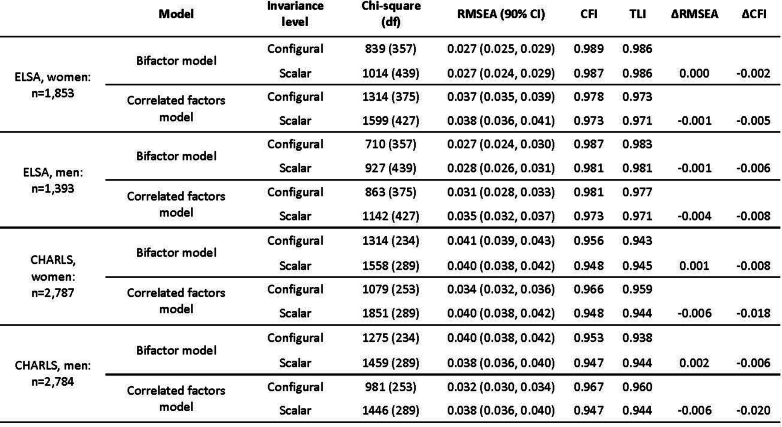
Measurement invariance testing in the sexes

CFI, comparative fit index; RMSEA, root mean square error of approximation; TLI, Tucker–Lewis index.

**Extended Data Table 6:**
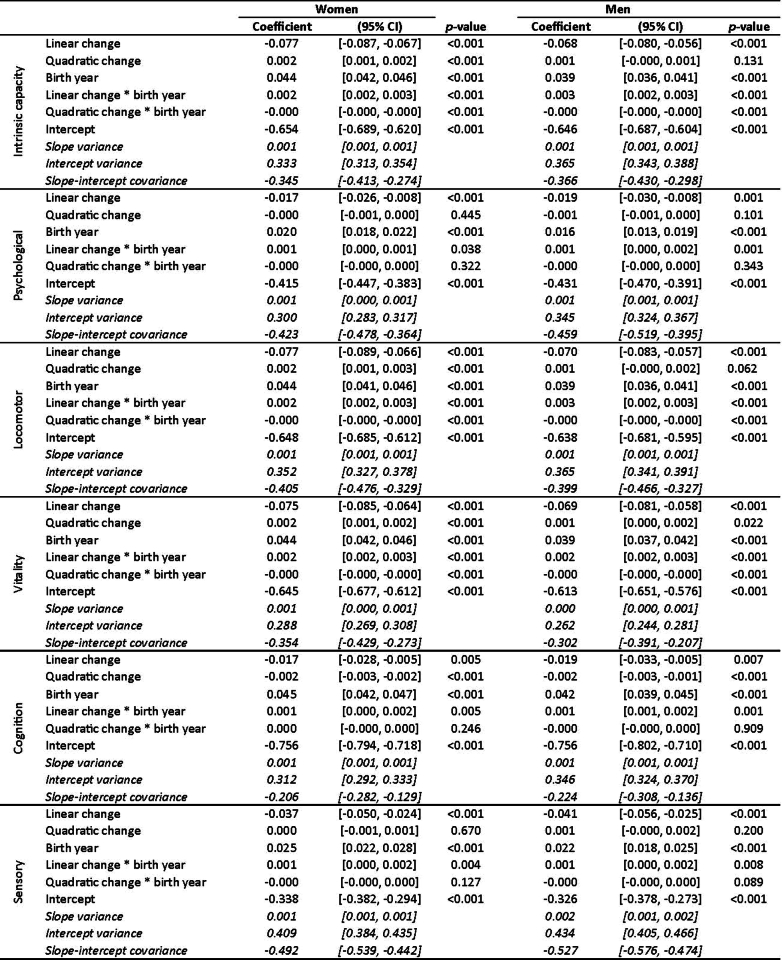
Results from multilevel growth curve models stratified according to sex in ELSA

A total of 7,841 women and 6,809 men from the ELSA waves 1 (2002) to 9 (2019) were used for the confirmatory factor analysis. Coefficients and 95% confidence intervals (CIs) were derived from bifactor models for intrinsic capacity and from the correlated factor models for each of the subdomains. Two-sided *P* values (unadjusted for multiple comparisons) were obtained, with *P* values below 0.001 presented as *P* < 0.001. Longitudinal weights were used to restore representativeness to participants aged 50 and older and living in England. Random effects are included in bold.

**Extended Data Table 7:**
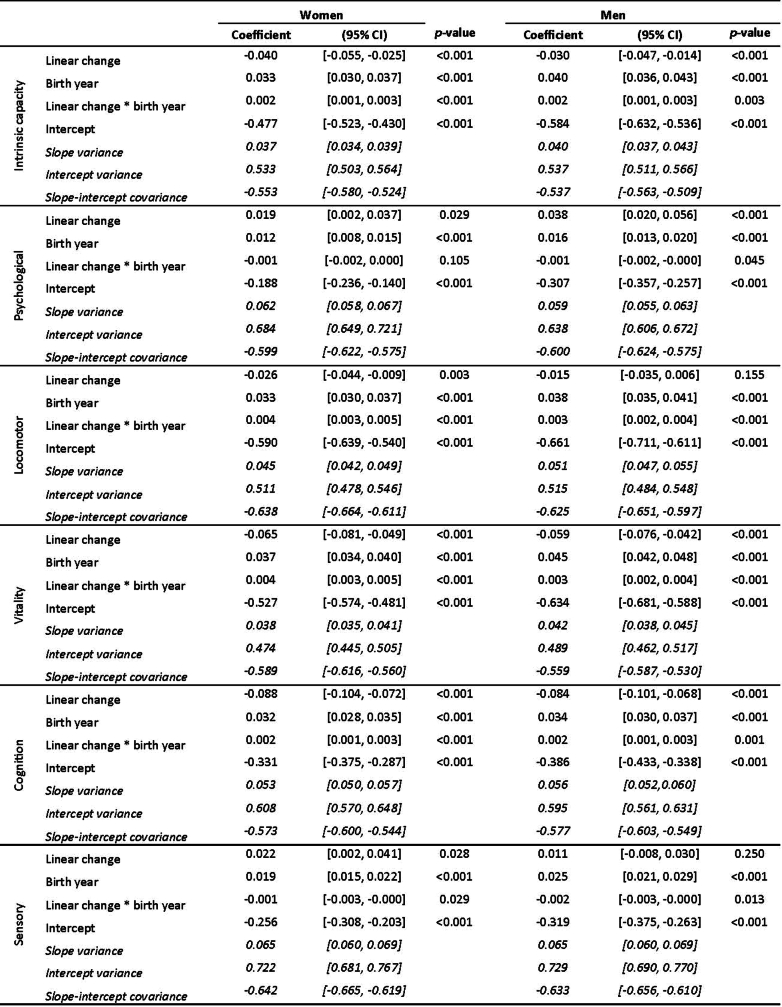
Results from multilevel growth curve models stratified according to sex in CHARLS

A total of 5,706 women and 5,705 men from the CHARLS waves 1 (2011) to 3 (2015) were used for the confirmatory factor analysis. Coefficients and 95% confidence intervals (CIs) were derived from bifactor models for intrinsic capacity and from the correlated factor models for each of the subdomains. Two-sided *P* values (unadjusted for multiple comparisons) were obtained, with *P* values below 0.001 presented as *P* < 0.001. Survey weights were used to restore representativeness to participants aged 45 and older and living in China. Random effects are included in bold.

## Data Availability

The data described in this article are available at the ELSA (https://www.elsa-project.ac.uk/) and CHARLS (http://charls.pku.edu.cn/en/) websites.

## Code availability

All code used in this analysis has been uploaded to the GitHub repository and is available at https://github.com/hanshi8860unsw/ELSA-CHARLS-IC-Cohort-Difference.
