# Supplementary material for: Cohort trends in intrinsic capacity in England and China
Source: Nat Aging. 2024 Dec 19;5(1):87–98. doi: 10.1038/s43587-024-00741-w (PMC11754101; doi:10.1038/s43587-024-00741-w)
Supplement: Supplementary file 1 — Measurement of intrinsic capacity indicators and additional analyses according to sex. [file 43587_2024_741_MOESM1_ESM.pdf]

nature aging

Supplementary information https://doi.org/10.1038/s43587-024-00741-w

Cohort trends in intrinsic capacity in England and China

In the format provided by the authors and unedited

Is 70 really the new 60? Cohort trends in functioning in England and China

SUPPLEMENTARY INFORMATION

Measurement of intrinsic capacity indicators

Longitudinal intrinsic capacity indicators were consistent with Beard et al. 2019 [1] and Beard et al. 2022 [2]. In order to make the measurement and analytical approaches as comparable as possible across CHARLS and ELSA, we focused on a set of common indicators that were used in both studies:

Locomotor subdomain indicators:

- *Walking speed:* In CHARLS and ELSA, each participant aged 60 and above was eligible for the timed walk test. In addition, prior to the actual test, participants were asked if they had any problems from recent surgery, injury, or other health conditions that might prevent them from walking. Only persons aged at least 60 years, willing to do the test and able to walk (walking aids were permitted) were asked to walk 2.5 meters in CHARLS (and 2.4 meters in ELSA) at their usual walking pace, twice. The time for both walks was recorded separately. In our analysis, we use the log-transformed time (seconds) of the two trials.
- *Chair-stand test:* The chair-stand test was used in CHARLS and ELSA as a measure of physical performance, assessed the time required to rise from a chair to a full standing position five times with arms folded across the chest, with slower times reflecting worse function. The test incorporated the use of the respondent's own armless, straight-backed chair. The time taken for the full stand was recorded in seconds both in CHARLS and ELSA. Participants were considered ineligible if they could not stand up without assistance; the use of walking aids, such as a walker or cane, was not permitted. The test was stopped if the person became too tired or short of breath, if the person used their hands, or if the nurse assessor felt concerned for the person's safety. In our analysis, we use the log-transformed time (seconds).
- *Balance:* Static balance was evaluated in ELSA and CHARLS through three separate tests, which formed part of the Short Physical Performance Battery [3]. Participants were ineligible for the tests if they were chair-bound or wheelchair-based; if it became clear after discussion that they were too unsteady on their feet; if they found it painful to stand; or if either the nurse assessor, or the participant, considered the test unsafe. We included data obtained for three components of the balance test: side-by-side stand (+1), semi-tandem stand (+1), and full-tandem stand (+1). A binary variable capturing whether the person was able to perform all these balance tasks or not was generated.
    - ➢ Side-by-side stand: Participants were asked to stand with feet together, side-by-side, for at least 10 seconds, using their arms, bending their knees or moving their body to maintain balance, but not moving their feet. If the participant was unable to hold the position for 10 s, one score was recorded, and no further tests were attempted. Those able to hold the position for 10 seconds scored an additional one and moved on to the semi-tandem stand;
    - ➢ Semi-tandem stand: Participants had to stand with the side of the heel of one foot, touching the big toe of the other foot for at least 10 s. Participants unable to hold the position for 10 s

scored nothing, and no further tests were attempted. Those able to hold the position for 10 s scored an additional one and moved on to the full-tandem stand;

- Full-tandem stand: For this test, participants had to stand with the heel of one foot in front of and touch the toes of the other foot. In CHARLS, those aged 70 and above and able to hold the position for at least 30 seconds were regarded as completed for this test, while those aged below 70 were required to hold the position for at least 60 seconds; In ELSA, all participants were required to hold the position for 10 seconds. Those able to complete the test score an additional one.

Vitality subdomain indicators:

- *Grip strength*: The grip strength test was used both in CHARLS and ELSA to test upper body strength. Handgrip strength (kg) of the dominant hand was assessed using a hand-held dynamometer. In the data set, there were two measures for each hand. An average of the dominant hand was calculated for analysis (if both hands were reported as dominant hand, we chose the larger measure). Any measurements carried out incorrectly or participants refused to perform the test were not included. Values above 60 kg were recoded into 60.
- *Forced expiratory volume (FEV):* Lung function was measured in CHARLS and ELSA using a spirometer. Eligible participants were asked to stand or sit, take a deep breath and blow into the spirometer as hard as they could. They were then required to repeat the procedure to give three technically satisfactory blows. The highest technically satisfactory measure of FEV was used in the analysis.
- *Blood assay:* The CHARLS and ESLA collected venous blood samples, and a complete blood count analysis was undertaken at local CDC laboratories (this included haemoglobin, haematocrit, white blood cell count, platelet counts, and mean corpuscular volume). Three tubes of venous blood were collected from each participant by medically trained staff from the China CDC, using a standard protocol. Detailed information on the technicalities of the blood analysis, the internal quality control, and the external quality assessment for the laboratory has been described on the website of CHARLS (http://charls.pku.edu.cn/index/en.html) and ELSA (https://www.elsa-project.ac.uk/). We used haemoglobin in this study for analysis.

Sensory subdomain indicators:

- *Hearing:* hearing status was assessed by asking participants to rate their hearing (using a hearing aid if they used one) as excellent, very good, good, fair, or poor.
- *Distant eyesight:* for distant vision, participants were also asked 'How good is your eyesight for seeing things at a distance, like recognizing a friend across the street (with glasses or corrective lenses if you wear them)'. Response options were categorized as (excellent/very good/good/fair–poor);
- *Near eyesight:* for near vision, 'How good is your eyesight for seeing things up close, like reading ordinary newspaper print'. Response options were categorized as above.

Cognition subdomain indicators:

- *Immediate recall:* based on the participant's ability to immediately recall a list of words.
- *Delayed recall:* based on the participant's ability to recall the same list of words four minutes later in CHARLS (and no minute specification in ELSA).
- *Time orientation/memory:* ability to accurately identify the current date (month, day and year).

Psychological subdomain indicators:

- *Affect and sleep:* Affect and sleep were assessed using the Center for Epidemiological Studies-Depression (CES-D) scale [6]. Items that were present in both ELSA and CHARLS (i.e., felt depressed, everything was an effort, sleep was restless, felt lonely, felt happy, and could not get going) were included. These items referred to how the participant felt and behaved during the last week. Each item corresponded with four categorized answers in CHARLS, rarely or none of the time (< 1 day), some or a little of the time (1 − 2 days), occasionally or a moderate amount of the time (3 − 4 days), and most or all of the time (5 − 7 days). In ELSA, the answer was recorded as dichotomous, 1-yes and 0-no.

References

1. Beard, J. R., Jotheeswaran, A. T., Cesari, M. & de Carvalho, I. A. The structure and predictive value of intrinsic capacity in a longitudinal study of ageing. *BMJ Open* **9**, (2019).
2. Beard, J. R., Si, Y., Liu, Z., Chenoweth, L. & Hanewald, K. Intrinsic Capacity: Validation of a New WHO Concept for Healthy Aging in a Longitudinal Chinese Study. *The Journals of Gerontology: Series A* **77**, 94–100 (2022).
3. Guralnik, J. M. *et al.* A short physical performance battery assessing lower extremity function: association with self-reported disability and prediction of mortality and nursing home admission. *Journal of Gerontology* **49**, M85–M94 (1994).
4. Lei, X., Hu, Y., McArdle, J. J., Smith, J. P. & Zhao, Y. Gender differences in cognition among older adults in China. *Journal of Human Resources* **47**, 951–971 (2012).
5. Lei, X., Smith, J. P., Sun, X. & Zhao, Y. Gender differences in cognition in China and reasons for change over time: evidence from CHARLS. *The Journal of the Economics of Ageing* **4**, 46–55 (2014).
6. Carleton, R. N. *et al.* The center for epidemiologic studies depression scale: a review with a theoretical and empirical examination of item content and factor structure. *PloS One* **8**, e58067 (2013).

Additional analyses by gender

In order to explore whether the results found varied by gender, we carried out an additional set of analyses accounting for gender (coded as "woman" or "man").

Measurement invariance testing across waves and genders

First, we investigated whether scalar invariance held across genders, extending the original measurement invariance testing (across waves) to include gender as well (across waves * genders). In ELSA, the two

lowest categories in the variable “memory” were grouped together as there was no observation with a zero score in the “wave 2, women” condition.

In ELSA, the estimation of the configural bifactor model led to convergence issues. The correlated factors model converged, but scalar invariance did not hold. Bifactor and correlated factors models could be estimated using CHARLS data, but scalar invariance did not hold. These results prevented us from meaningfully comparing the resulting factor scores across gende and over time in both ELSA and CHARLS.

In light of these results, we proceeded to investigate whether scalar invariance held over time *within* genders, which may enable meaningful comparisons of the intrinsic capacity and respective subdomains’ levels over time in the genders in which such invariance was found to hold. The results of this measurement invariance testing are provided in **Extended Data Table 5**. In line with the results from the overall analyses, scalar invariance held in all cases except for the correlated factors model in CHARLS, where the loss in fit was acceptable based on the RMSEA but larger than the usual threshold based on the CFI. As scalar invariance held within genders but not across them, factor scores were derived for each gender separately.

Trajectories of intrinsic capacity and subdomains by gender

Multilevel growth curve models were estimated by gender, using the factor scores derived from the corresponding models. The multilevel models’ coefficients are detailed in **Extended Data Table 6** (for ELSA) and **Extended Data Table 7** (for CHARLS). To aid interpretation, the visual depictions of the different trajectories are provided in **Extended Data Figure 1** for ELSA, and in **Extended Data Figure 2** for CHARLS by calendar year and age, respectively.

Despite differences in some of the model coefficients across genders, the similarities in the trajectories within genders are clear from their visual representation, which in turn are very similar to the trajectories obtained from the main models not stratified by gender. Perhaps the most noticeable difference lays in the psychological subdomain in CHARLS, where men increase more rapidly over time than women; however, similar to the main pooled analyses, comparisons of the subdomains’ levels over time are limited and should be made with caution even within genders due to the lack of longitudinal invariance.
